# Obesogenic environments and obesity: a comment on ‘Are environmental area characteristics at birth associated with overweight and obesity in school-aged children? Findings from the SLOPE (Studying Lifecourse Obesity PrEdictors) population-based cohort in the south of England’

**DOI:** 10.1186/s12916-020-01538-5

**Published:** 2020-03-18

**Authors:** Matthew Hobbs, Duncan Radley

**Affiliations:** 1 Health Sciences, University of Canterbury, Private Bag 4800, Christchurch, 8140 New Zealand; 2 Applied Obesity Research Centre, Leeds Beckett University, Leeds, LS6 3QT UK

**Keywords:** Health geography, Obesity, Environment, Greenspace, Cohort study

## Background

Since 1980, the prevalence of obesity has doubled in more than 70 countries [1]. Worldwide, in 2015, the prevalence of children and adults with obesity was 5 and 12%, respectively. This equates to 107.7 million children and 603.7 million adults [1]. The physical and psychological consequences of obesity are well documented, including an increased risk of type 2 diabetes, adverse cardiovascular outcomes, discrimination and reduced self-esteem. Moreover, it was estimated that obesity accounted for approximately 4 million deaths and 120 million disability-adjusted life-years worldwide in 2015 [1].

The relationship between our health and the environment or places in which we reside and work, day to day, dates back centuries. It was Hippocrates who first argued that health was a product of environmental factors and highlighted a need for harmony between the individual, social and natural environment. Fast-forward to the present day and the term ‘obesogenic environment’ has been coined to refer to the influences that the surroundings, opportunities or conditions of life have on promoting obesity in individuals and populations [2]. While the causes of obesity are complex and obesity is multifaceted in aetiology, it is plausible that the condition is driven largely by environmental factors, which undermine the self-regulatory capacity that people have to make responsible decisions about personal diet and physical activity [3]. For instance, it is likely that the increased availability, accessibility and affordability of energy-dense foods, along with intense marketing of such foods, are examples of such environmental factors that, at least partly, explain excess energy intake and weight gain [4].

## The current state of evidence

In recent decades, many researchers have attempted to unpick the relationship between physical and social environment and how these affect people’s weight status. However, the evidence base is inundated with different approaches, methods, metrics and environmental variables, making comparison between studies difficult [5], the search for unequivocal evidence elusive and the translation of evidence into policy near impossible. Highlighted in a recent systematic review of 113 studies, null associations dominated across all measurement methods, comprising 76% of 1937 associations in total [5]. The accompanying comprehensive appraisal of study quality indicated that, in general, study quality and methodological reporting were poor and study findings were at risk of bias. For instance, only three of the included papers (2.7%) contained all relevant details on how food outlets were geocoded. In addition to these methodological limitations, current evidence often relies on static definitions of exposure; for instance, around a participant’s residential home address [6, 7]. This is now well known as the ‘Uncertain Geographic Context Problem’. Recently, Zhao et al. [7] showed that the contextual areas used to derive a particular environmental variable affect whether or not an environmental variable has a significant influence on participants’ body mass index. Indeed, using global positioning systems to track movement has indicated that research often assumes that children and adults are less mobile than they really are. Further, most studies investigating links between environmental factors, such as fast-food outlets and obesity, are cross-sectional [5], making any causal associations unclear.

## The SLOPE study

The paper presented by Wilding et al. [8] provides a unique and important longitudinal addition to current evidence. Using a population-based cohort in the south of England, the authors examine how environmental characteristics, including greenspace, walkability, supermarket density, unhealthy food outlet relative density, spaces for social interaction and air quality at birth are associated with overweight and obesity in school-aged children (14,084 children aged 4–5 years and 5637 aged 10–11 years). It is particularly important to consider these age groups of children given recent evidence from the English 2018/19 National Childhood Measurement Programme, which showed 9.7% of reception class children (aged 4–5 years) were obese, while the prevalence of obesity in year 6 children (aged 10–11 years) was 20.2% [9]. This use of a large dataset of routine data reduces the risk of sampling bias and increases the power to detect meaningful associations between exposure and change in the outcome. Of note, the authors assigned area characteristics on an annual basis (except for walkability) to maximise the relevance of estimated exposures. This considerable methodological effort should be commended.

The use of longitudinal data should also be recognised, since many research studies can only aspire to such longitudinal exposure measures. Furthermore, by conducting subanalysis of children who moved home lower super output area between birth and weight measurement, the study was strengthened by accounting for population migration. This is important because longitudinal designs can address residential self-selection bias by establishing temporality [10]; accounting – for instance – for residential relocation that may be triggered by events such as marriage or employment changes, which may also influence health-related behaviours and subsequent health outcomes [10]. The study concludes that increased access to greenspace and the subsequent protection of greenspace may have a role in early prevention of childhood obesity. While effects were often small, these are likely to be meaningful in effect size when considered at a population level. Future research may wish to consider confounders that the authors recognised, but were unable to adjust for, including paternal factors, maternal diet in pregnancy, parental diet and physical activity, family income, child’s diet and physical activity.

## Conclusions

While there is much work to be done to better understand how the environment in which we reside and work affects both our behaviours and health, Wilding et al. (8) provide an important contribution to the current state of evidence. There continues to be growing interest in the environmental determinants of health outcomes and health behaviours. However, as we outline, there are several notable strengths that can be taken from this article in future research. We hope these strengths are recognised and will be considered and incorporated in the development of future research, where feasible.

## Data Availability

Not applicable.
